# UAV Positioning Mechanisms in Landing Stations: Classification and Engineering Design Review

**DOI:** 10.3390/s20133648

**Published:** 2020-06-29

**Authors:** Musa Galimov, Roman Fedorenko, Alexander Klimchik

**Affiliations:** Innopolis University, Center for Technologies in Robotics and Mechatronics Components, 420500 Innopolis, Russia; m.galimov@innopolis.ru (M.G.); a.klimchik@innopolis.ru (A.K.)

**Keywords:** charging station, charging, docking station, landing platform, landing, positioning, UAV, vertical take-off and landing

## Abstract

Landing platforms’ automation is aimed at servicing vertical take-off and landing UAVs between flights and maintaining their airworthiness. Over the last few years, different designs for the landing platforms have been proposed. This shows a strong development and establishment of automatic landing platforms with UAV positioning devices on the landing site. Positioning and safe fixation of the UAV are some of the main features of the landing platform, especially if it is mounted on a movable vehicle. This article focuses exclusively on the landing platform and its elements that provide the positioning of the UAV by affecting it during and after the landing. Both active devices and mechanisms and passive elements used for positioning are considered. This article, based on the review of recent patents and publications, gives the classification of positioning approaches used in landing stations with the analysis of the required landing precision, as well as the pros and cons of each type of approach.

## 1. Introduction

With a dramatic growth in the UAV market in recent years, the amount of research efforts dedicated to UAVs increased. However, the short battery life essentially restricts the applications of unmanned aerial vehicles and has proven to be a hard issue to tackle [1]. To overcome this issue, several approaches have been proposed. Approaches aimed at improving the power supply include the development of batteries with high-power density, hydrogen fuel cells, using internal combustion engines and hybrid power schemes, and solar panels. These approaches still have drawbacks, including the high cost and high weight and/or dimensions of power supplies. Another approach is using a tether for power and data, which could give almost limitless flight duration, but limits the range and maneuverability of the UAV, while also making the system much more expensive. Vertical Take-Off and Landing (VTOL) planes are effective in fixed wing cruise flight and still have the ability to take off and land vertically as multi-rotor UAVs, but using wings makes UAVs larger, which is not always applicable.

Landing platforms for UAVs are among the most promising approaches to extend their operation duration with minimum vehicle modifications. Moreover, in addition to the battery charging or replacement function, automatic landing platforms could perform other functions, such as picking up or loading the cargo, data exchange and processing, etc. [2]. Landing platform applications include: continuous mapping and survey; security; delivery of medicine, food items, groceries, parcels delivery, and others. Landing stations could also be mounted on ground or surface vehicles and used as an interface for group interaction between heterogeneous vehicles [2,3,4,5,6,7].

Landing platforms’ automation is aimed at servicing vertical take-off and landing UAVs between flights and maintaining their airworthiness. These tasks include:

- connecting and disconnecting contacts to charge the batteries;
- exchanging information with a UAV;
- battery replacement/refueling;
- loading and unloading of transported goods, and so on.

Each of these operations, when performed automatically, requires precise positioning and safe fixation of the UAV on the landing platform, especially if the landing platform is used on a movable vehicle. The process of the positioning of the UAV at the desired point on the landing pad begins while the UAV is in the air. At this stage, the UAV usually adjusts its position to land at a given point. The deviation from the desired landing point of the UAV depends on the method and sensors chosen to detect the landing location, the performance of the UAV control system, and the meteorological conditions, especially visibility, precipitation, and gusty winds. It should be noted that, at the last moment of landing, the UAV usually turns off the propeller motors and descends uncontrollably to the landing platform. This also contributes to a deviation from the desired landing point. Further positioning can be accomplished by influencing the landing platform elements on the UAV during and after the full landing of the UAV on the landing platform. This article does not cover the positioning of the UAV in the air, but focuses exclusively on the landing platform and its elements providing the positioning of the UAV by affecting it during and after the full landing.

Many works present particular designs of landing stations [1,7,8,9,10] and their parts, such as charging devices [1,11,12,13,14]. There is growing interest in the application of multiple UAV landing stations and their efficient placement and operation [15,16,17]. A huge number of articles are dedicated to the task of UAV landing on moving platforms, which has been in particular accelerated by the Mohamed Bin Zayed International Robotics Challenge (MBZIRC) [2,18,19,20,21]. Most of the works on landing are dedicated to control [22,23,24,25] or localization and computer vision for UAV positioning in the landing station [26,27,28,29,30,31].

Meanwhile, landing station construction itself should be considered for effective control of the landing process. It defines the requirements for sensor system accuracy, as well as the landing sequence algorithm and the moment of the motors turning off. Positioning mechanisms of landing stations accomplish further positioning of the UAV by influencing it during and after the full landing. To the best of the authors’ knowledge, there is no survey article that has introduced UAV landing platforms’ designs and classification for researchers and engineers in this area. This article is aimed to fill this literature gap.

The paper objective is to give a comprehensive review and classification of UAV landing stations by their positioning device construction with the analysis of the required landing precision, as well as the pros and cons of each type of approach. The main novelty and contributions of this work are:

- a review of the state-of-the-art of UAV landing stations based on patents and commercial prototypes and products;
- a classification of approaches for UAV positioning in landing platforms;
- an analysis of the required landing precision, as well as the pros and cons of each type of approach.

The graphic material presented in this article is mostly borrowed from the cited sources while retaining the original numbering of elements. In the article, there are designations only of those elements that are relevant to the topic considered.

## 2. Classification of Approaches for UAV Positioning in Landing Platforms

The choice of the types of positioning devices in the landing platforms is primarily based on the requirements for battery charging or replacement, fixing the UAV in the landing platform, loading and unloading the UAV cargo, etc. The operating conditions considered should include weather conditions and stationary or mobile usage. The technique and performance (in particular, the accuracy) of the UAV landing is also significant for the choice of types of positioning devices.

**Figure 1:**
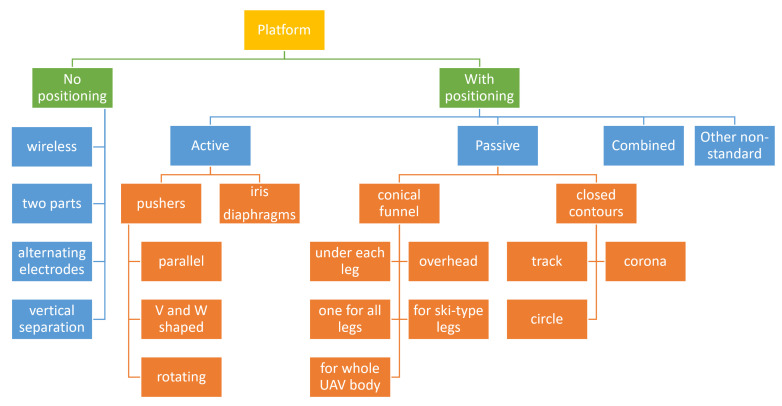
Classification of approaches for UAV positioning in landing platforms.

**Figure 2:**
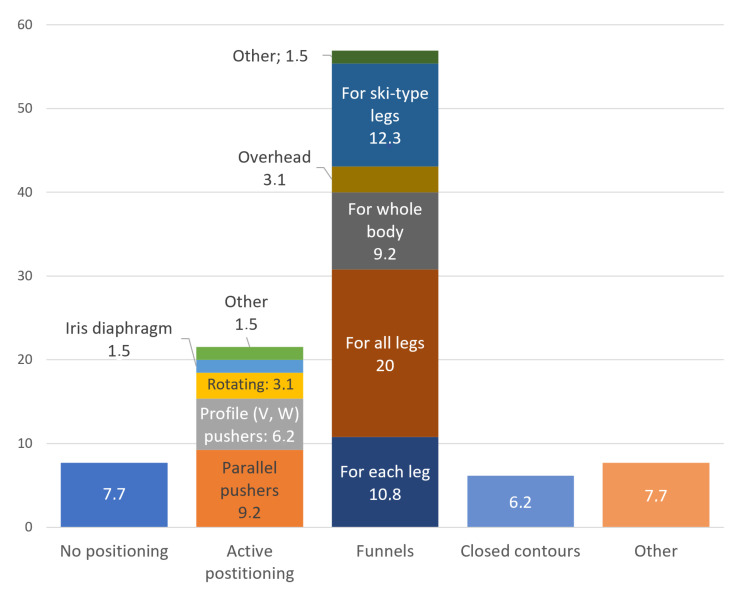
The percentage of the usage of the positioning devices of certain types determined during the patents’ analysis.

The classification of landing platforms by types of positioning devices presented in Figure 1 and the percentage of the usage of positioning devices of certain types in Figure 2 are based on the analysis of the patents of landing platforms in which the designs of positioning devices are revealed. We used Google Patents as the search engine, which indexes patents and patent applications. Patents and patent applications relevant to the design of positioning devices formed a database for further analysis in this article. All patents selected are listed in the References. We consider the constructions of different types and discuss their advantages and disadvantages and preferences in choosing them.

The landing platforms are divided into platforms without positioning devices, platforms with active positioning devices, platforms with passive positioning devices, platforms with a combination of positioning devices, and platforms with non-standard positioning devices.

For platforms without positioning devices, the UAV lands at the landing site with the accuracy provided by the UAV control system. The UAV service devices that are part of the landing platform should be able to service the UAV at the position it stands after landing. Typically, this is an electrical connection via large contacts. No other manipulations are performed in this case.

Platforms with active positioning equipment include mechanisms and devices that move the UAV to the required position and turn it in the required direction. The positioning mechanisms are selected according to the accuracy and speed requirements of the UAV’s positioning. Platforms with active positioning equipment include various types of pushers.

For platforms with passive positioning equipment, there are no mechanisms or devices to move the UAV: the horizontal movement of the UAV for positioning during a landing is provided by transforming its vertical motion. This can be the movement over a specially designed inclined surface that leads the UAV to the desired positioning point. The UAV positioning process is completed when the UAV has landed. Passive positioning devices include funnels, slopes, inclined edges, etc.

Each of the above devices has its own features and limitations. Platforms with a combination of positioning devices of different types can achieve better results by taking advantage of each method.

Platforms have been categorized as platforms with non-standard positioning devices, typically in the case of unusual landing devices or positioning methods that are not listed above. However, they can be active, passive, and combined.

Each type of positioning equipment can be divided into sub-types, which are shown in Figure 1 and further explained. The percentage of the usage of the positioning devices of certain types determined during the patents’ analysis is as follows (Figure 2):

- platforms without positioning devices 8%
- platforms with active positioning device 22%
- platforms with passive positioning device 63%
- platforms with non-standard positioning devices 7%.

Platforms with combinations of different positioning devices were not considered in this distribution. As shown, passive and active positioning devices are most commonly used. Among these types, the most frequently used are:

- positioning devices with parallel pushers (42%) and profile pushers (28%) among active positioning devices
- a positioning funnel for all UAV legs (39%) among the passive positioning devices.

## 3. Landing Platforms without UAV Positioning

The most straightforward approach for recharging the batteries is to make electrical contacts on the UAV landing gear and the landing site. The landing site is divided into electrical contact zones isolated from each other. In the simplest case, the landing site can be divided into two equal parts of different polarity with rectangular electric contacts. The UAV, when landing, touches the electrical contacts of the landing site by its contacts on the landing gear (legs). Such a landing platform requires the UAV to land with a precision equal to half of the contact side length.

**Figure 3:**
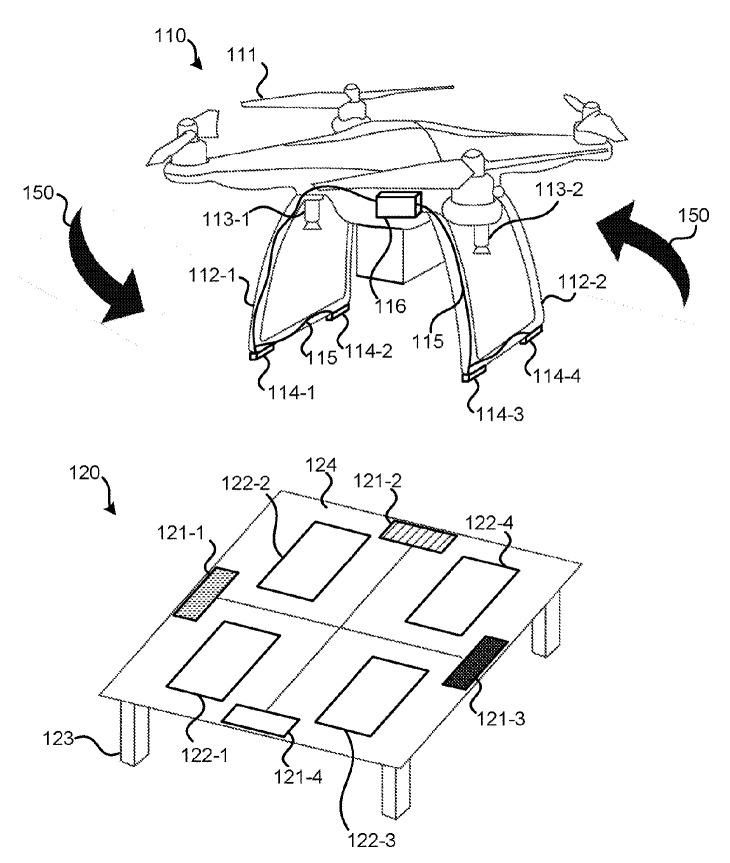
Unmanned aerial vehicle docking system [103] as an example of a landing station without positioning. 110, UAV; 112-1 ... 112-4, UAV legs; 114-1 ... 114-4, electric contacts on UAV legs; 122-1 ... 122-4, electric contacts on the landing site; 124, landing site.

Alternating electrodes of different polarity in the form of strips [32] or in the form of four [33,34] of more [35] squares or rectangles of chessboard type can be installed on the landing site. If the number of contacts is two or four, the UAV must be able to perform accurate landing, allowing each landing leg to sit on its own contact on the landing platform. Figure 3 shows a system of unmanned aerial vehicle docking [34] with four electric contacts 122-1 ... 122-4 on the landing surface 124 and electric contacts 114-1 ... 114-4 on UAV landing legs 112-1 ... 112-4.

The large number of contacts eliminates the requirement for the accuracy of the UAV landing. Regardless of where the UAV support lands, it is not possible to land two UAV legs of different polarities on one contact. Once the UAV has landed, the polarity matching of the electrical contacts on the UAV legs and the landing site is performed. The electrical contacts of the UAV legs can be adjusted for the electric contacts of the landing site [32] or vice versa [35].

**Figure 4:**
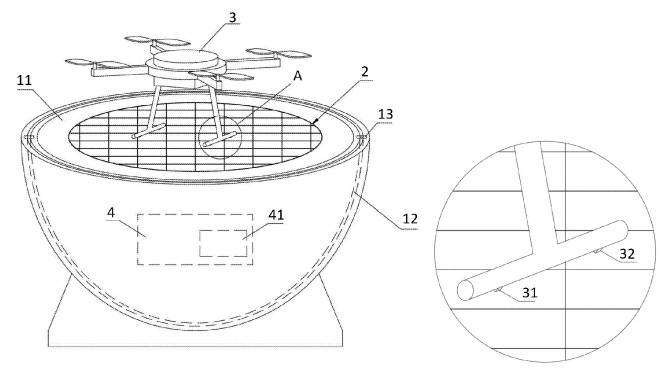
Automatic contact-type UAV charger on a landing station [35]. 2, landing surface with electric contacts; 3, UAV; 4, charging controller; 31, 32, electric contacts on UAV legs.

Figure 4 shows an automatic contact-type UAV charger on a landing station [35] with a set of electric contacts 21 on the landing area 2 and electric contacts 31 and 32 on the UAV legs.

**Figure 5:**
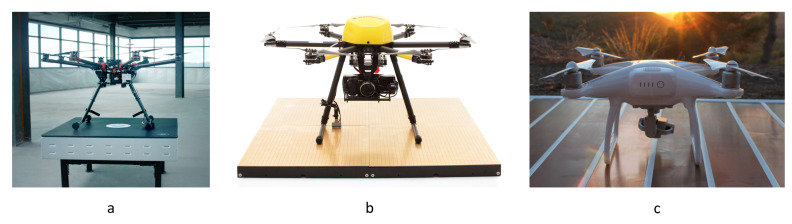
Examples of commercially available UAV charging platforms without positioning. (**a**), wireless charging pad by WiBotic (Seattle, WA, United States) [37]; (**b**), landing pad with alternating electrodes and special retrofit kit on the landing gear by Skysense, Inc. (San Francisco, CA, United States) [38]; (**c**), landing pad with alternating electrodes by Edronic (Cartagena, Murcia, Spain) [39].

Figure 5 shows examples of commercially available UAV charging platforms without positioning with a wireless charging pad (a), alternating electrodes and a special retrofit kit on the UAV landing gear (b), and a landing pad with alternating electrodes (c).

**Figure 6:**
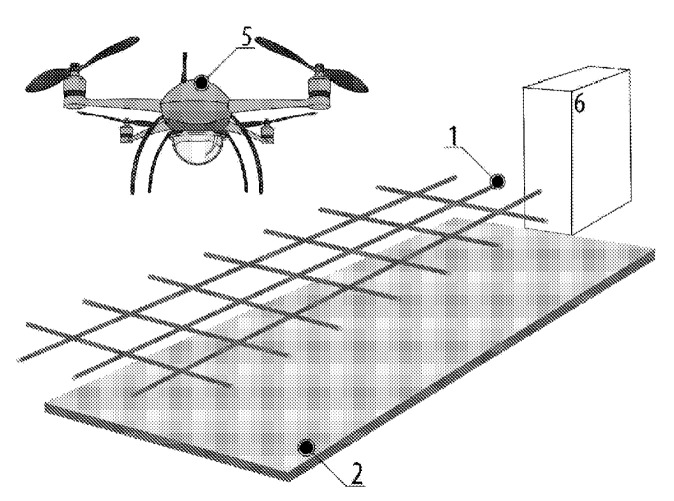
Method of recharging the batteries of unmanned aerial vehicles [36] as an example of a landing station without positioning with vertical electrodes separation. 1, electrode in the form of a metal grid; 2, electrode in the form of a metal sheet; 5, UAV; 6, charging controller.

Figure 6 [36] shows the method of charging the UAV’s batteries with vertical separation of electric contacts. The charging device consists of two electrodes: the upper one is made in the form of a metal grid 1 and is installed above the landing pad 2, which is made of an electroconductive material (metal sheet). UAV 5 has two electrodes: one is located at the ends of the legs and is freely passing through the cells of the upper electrode of the charging unit, and the other is located on the hull of the aircraft. When the UAV lands on the landing site, there is an electrical contact between the upper electrode and the UAV’s body and the lower electrode and the UAV’s legs. This landing site design also ensures that the UAV is charged regardless of the accuracy of the landing.

**Figure 7:**
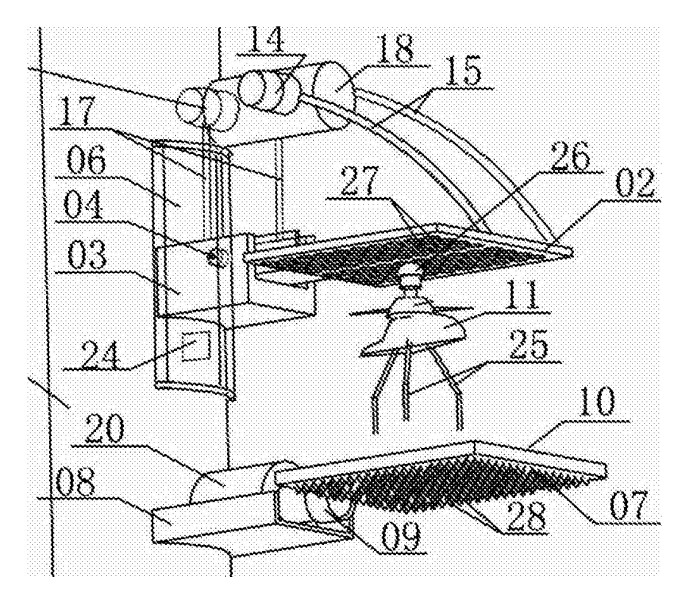
UAV charging system [40]. 02, upper electrode; 10, landing pad; 11, UAV; 25, UAV legs (lower electrodes); 26, UAV upper spherical electrode.

In the system of the UAV charging platform installed on the streetlamp [40] (Figure 7), the method of the vertical separation of electrodes is also applied. Electrodes of different polarities are on legs 25 of UAV 11 and on the spherical joint above the UAV body 26. The UAV lands on the landing pad 10, which has an electrode of one polarity. After that, the upper electrode 02 of the opposite polarity is lowered on it. The dimensions of the landing pad 10 and the upper electrode are equal. The dimensions of the landing platform should be equal (or greater than) to the overall dimensions of the UAV chassis with the permissible deviation of the UAV position. If it is required to use an enclosure wall for the landing site, the dimensions of the UAV with propellers should be considered. This condition is also true for most types of landing platforms discussed below.

Landing platforms of this type are pretty simple, but have certain disadvantages. The main disadvantages are that the UAV is not fixed at the landing site and the electrical contacts are not fixed. In the event of a landing site vibration or a wind gust, the UAV may move out of position and lose its electrical contact, or the UAV may land on one electrical contact with opposing polarity contacts. This is most dangerous for landing platforms with a small number of contacts. For this reason, this type of landing platform is not used on mobile vehicles.

## 4. Active Methods of UAV Positioning

### 4.1. Active Method of UAV Positioning with Parallel Pushers

**Figure 8:**
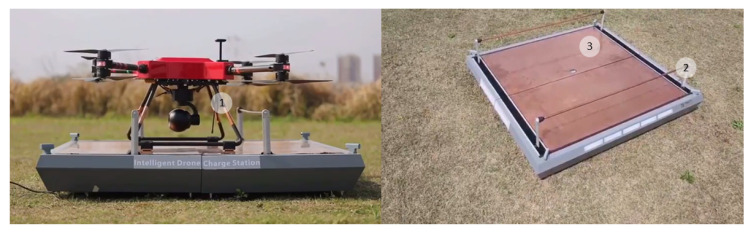
Ewatt Aerospace’s [46] Smart Charging UAV Platform without enclosure walls. 1, UAV leg with electrodes; 2, landing site; 3, electrodes.

The active methods of positioning of the UAV after landing on a landing site are widely used. The most frequently used method is one or two pairs of pushers parallel to each other and working synchronously, moving the UAV by its legs to the center of the landing site [41,42,43,44]. By means of these pushers, the UAV legs can be led to the contacts located on the landing site, providing electrical connection. Pushers can contain elements of fastening of UAV legs to the landing platform [42,43,44], as well as electric contacts [41,42]. In the autonomous station [45] by Boeing Co., only two pushers are used. They lead the UAV to the edge of the landing platform where the battery replacement device is installed. Ewatt Aerospace’s [46] Smart Charging Platform shown in Figure 8 has a landing site and moving electrodes, contacting with electrodes on the UAV legs. The UAV leg has electrodes on the top and bottom.

**Figure 9:**
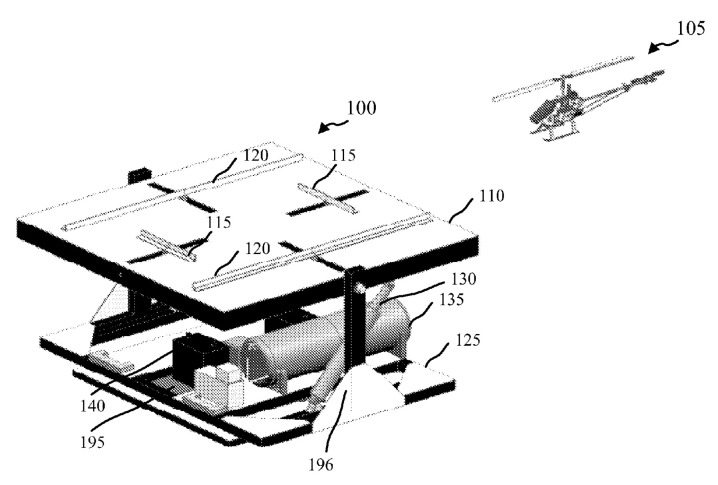
Unmanned aerial vehicle docking station [43] with parallel pushers for UAV positioning by the University of Denver. 105, UAV; 110, landing site; 115, longitudinal pushers; 120, lateral pushers.

Figure 9 shows a docking station of an unmanned aerial vehicle by the University of Denver [43]. Landing site 110 contains a pair of longitudinal pushers 115 and a pair of lateral pushers 120. Each pair of pushers has synchronous actuators providing movement of the landed UAV 105 to the center of landing site 110. The landing area 110 can be used to mount the UAV 105 fasteners or electric contacts.

**Figure 10:**
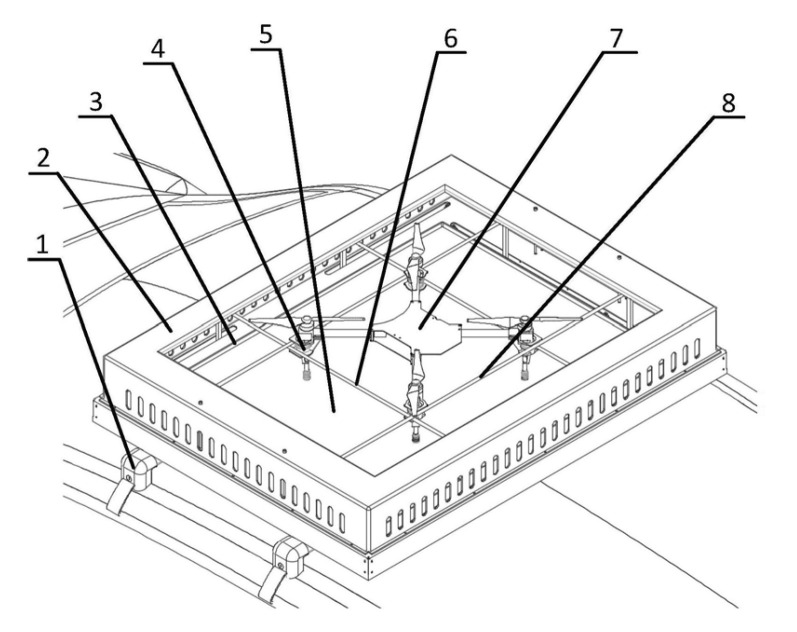
Unmanned aerial vehicle landing platform mounted on a mobile vehicle [44] with parallel pushers for UAV positioning by Beijing Institute of Technology. 4, UAV fixing element; 5, landing site; 6, short clamping bar; 7, UAV; 8, long clamping bar.

The landing platform for an unmanned aerial vehicle [44] (Figure 10) by Beijing Institute of Technology also contains two pairs of pushers (clamping bars 6 and 8), but the fixing of the vehicle is made by the same pushers with specially placed fixing elements 4 on the body of UAV 7. This solution allows for quick fixation of the UAV after its positioning and is suitable for use in landing platforms mounted on mobile vehicles.

**Figure 11:**
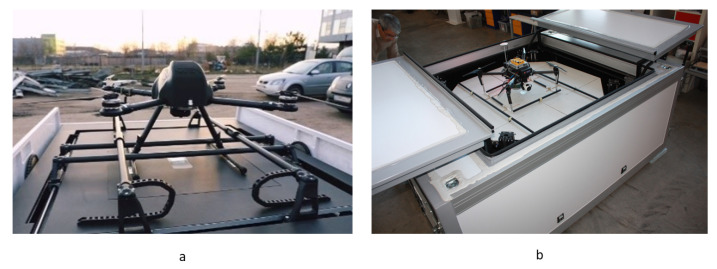
Examples of commercially available platforms for UAVs with 4 parallel pushers by: (**a**), COEX [47]; and (**b**), Innopolis University [48].

Examples of commercially available platforms for UAVs with 4 parallel pushers by COEX [7] and Innopolis University are shown in Figure 11.

**Figure 12:**
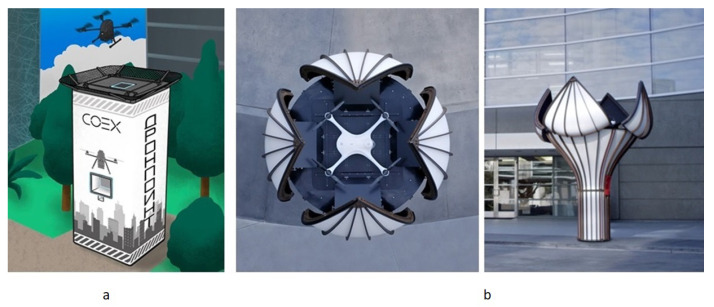
Examples of commercially available platforms for UAVs with parallel pushers with cargo unloading by: (**a**), COEX [47]; and (**b**), Matternet [49].

Since positioning systems with parallel pushers are the most straightforward and popular approach, the first commercial delivery platforms (Figure 12) with unloading of cargo are offered on the basis of this approach.

### 4.2. Landing Platforms with “V”- and “W”-Shaped Pushers

**Figure 13:**
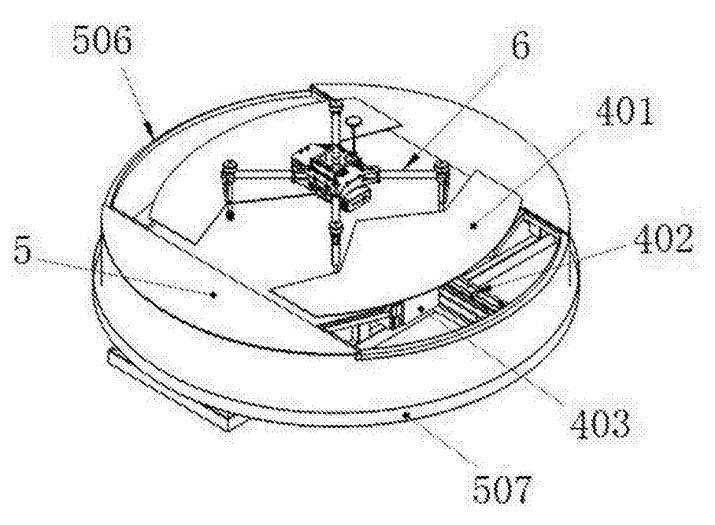
Charging platform for unmanned aerial vehicle [51] with W-shaped pushers for positioning. 6, UAV; 401, pushers with shaped edges.

Reducing the number of pushers simplifies the design of the landing platform. This is made possible by the use of shaped pushers. Pushers with “W”-shaped edges facing each other are used for positioning of the UAV with vertical legs [50,51]. Figure 13 presents the auto-charging platform for an unmanned aerial vehicle [51]. The device contains pushers 401 with “W”-shaped edges. In the synchronous motion of these pushers, the UAV 6 legs slide along the edges of the corners and end up in the inner corners. This ensures correct positioning both in the direction of movement of the pushers and in the orthogonal direction. Electrical contacts and/or grips can be installed at the tops of the inner corners of the pushers 401 to fix the UAV after positioning.

**Figure 14:**
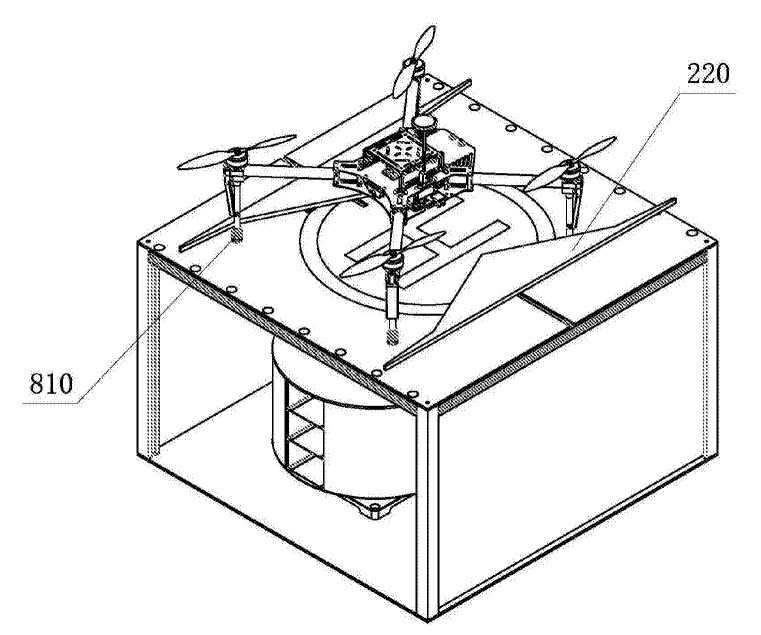
Landing platform with battery changer and V-shaped pushers for positioning [52]. 810, UAV leg; 220, pushers with V-shaped edges.

The same result is achieved in the landing platform with the device for batteries replacement [52] by Shan Dong University, in which “V”-shaped pushers 220 are used (Figure 14). When the pushers 220 move towards each other in the process of positioning, the central ledge of the pusher 220 pushes the UAV’s legs 810 across its movement and thus positions the UAV both in the forward and perpendicular direction to its movement. In the patent [53] by Coretronic Intelligent Robotics Corp, the “V”-shaped pushers are moving simultaneously with the the doors of the landing station driven by the same actuator.

To reduce the number of pushers, special forms of UAV legs are also used. For example, in the platform [54] by Nanjing Information Engineering University, the UAV landing gear is hexagonal and contains electrical contacts. Positioning pushers are made with “V”-shaped ledges with an angle of 120deg containing corresponding contacts.

**Figure 15:**
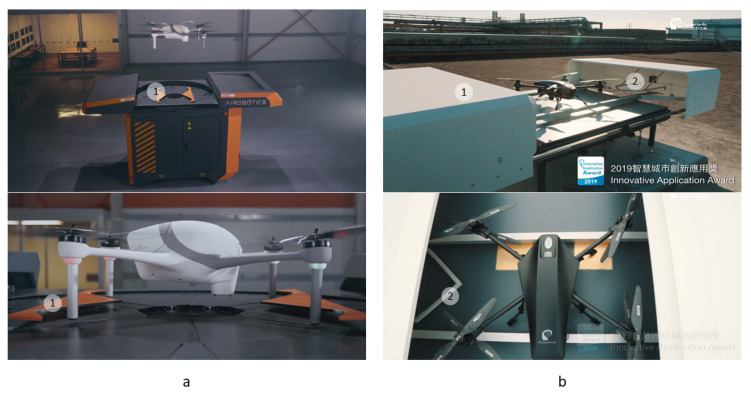
Examples of commercially available platforms for UAVs with V- and W-shaped pushers. (**a**), Airobotics [55] station with parallel W-shaped pushers (1); (**b**), Coretronic Intelligent Robotics (CIRC) [56] station with V-shaped pushers (2) integrated with doors (1).

Examples of industrial models of landing platforms for UAVs with “V”- and “W”-shaped pushers from Airobotics [55] and Coretronic Intelligent Robotics [56] are shown in Figure 15.

### 4.3. Landing Platforms with Rotating Pushers

**Figure 16:**
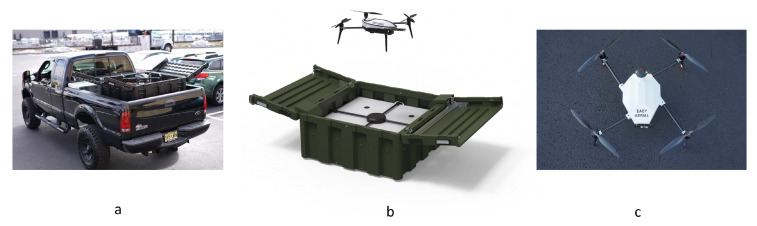
Example of a commercially available landing platform with rotating pushers (**a**,**b**) for a UAV with eccentric beams (**c**) by Easy Aerial [58].

**Figure 17:**
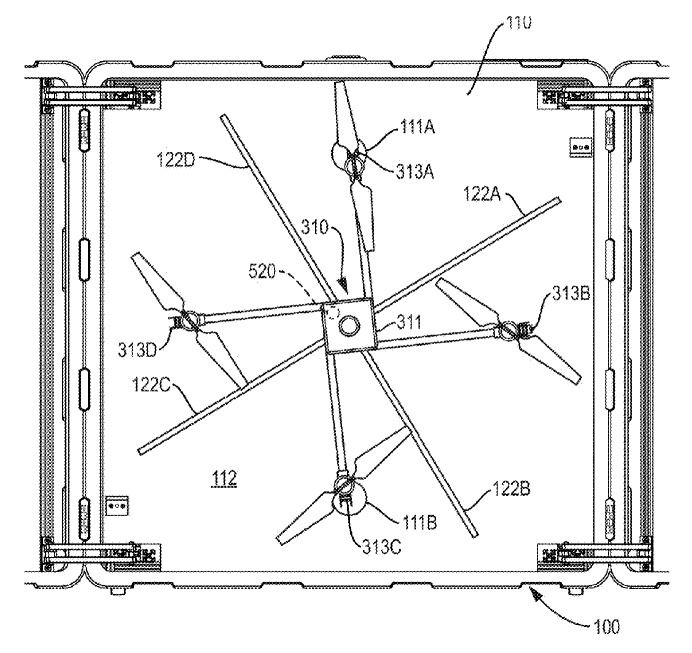
Charging station for unmanned aerial vehicles with a rotating pusher centering mechanism by Easy Aerial Inc. [59]. 110, landing surface; 111a, 111b, electrical contacts on the landing surface; 112, electrical insulating material of the landing surface; 122a, 122b, 122c, 122d, bars of the pusher; 310, UAV; 311, UAV body; 313a, 313b, 313c, 313d, UAV legs.

**Figure 18:**
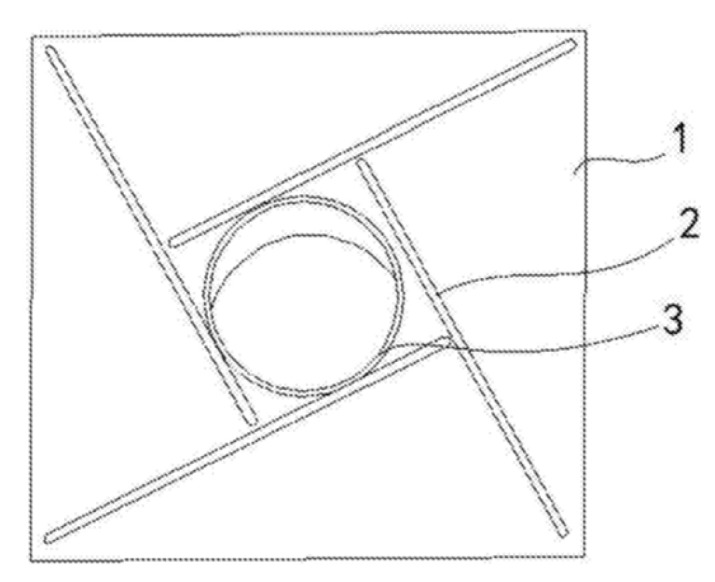
Landing platform with rotating pushers [57]. 1, landing platform; 2, rotating pushers; 3, UAV.

The next type of pushers is rotary pushers (Figure 16 and Figure 17). The platform [57] by Beijing Honeycomb Agricultural Science and Technology Co., Ltd. (Beijing, China), has rotary pushers installed on vertical shafts at the corners of the landing site (Figure 18). The length of rotary pushers 2 is shorter than the side of the landing platform 1. Therefore, when simultaneously rotating inwards of the landing platform direction, their ends come across the neighboring pusher, forming a square in the center of the landing platform. The description [57] provides a calculation of the pusher’s bar lengths required for normal positioning of the UAV.

The original device for positioning of the UAV in the charging station with a centering mechanism, shown in Figure 16 and Figure 17, was proposed in [59] by Easy Aerial Inc. (Brooklyn, NY, United States). The device is intended for landing and positioning of multirotor UAVs with special legs. The UAV legs 313 are located on beams, which in their turn are installed on UAV body 311 eccentrically. In the middle of the landing area, there is a rotating actuator with a vertical shaft, on which a rotating pusher 122 with bars is mounted. The number of pusher’s bars is equal to the number of UAV legs 313. After the UAV has landed, the vertical shaft begins to rotate, and the pusher’s bars make the UAV rotate by pushing its legs. The interaction of the UAV legs with the landing surface and the pusher and the eccentricity of the UAV beams mount causes the center of the UAV to move towards the center of the vertical shaft. Rotation stops when the UAV’s legs stand on the electrical contacts 111 on the landing surface.

### 4.4. Landing Platforms with Iris Diaphragms

**Figure 19:**
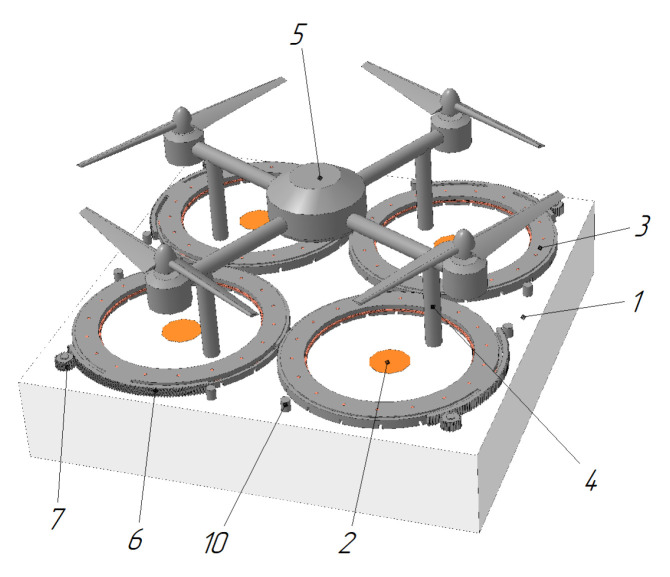
UAV landing platform with iris diaphragms for positioning [60] by Innopolis University. 1, landing surface; 2, electrical contacts on the landing surface; 3, iris diaphragm; 4, UAV legs; 5, UAV.

In fact, iris diaphragms are a special kind of rotating pusher under each foot of the UAV when used for positioning. However, due to its design originality, it has been classified as a separate subtype of active positioning systems. The landing platform for vertical take-off and landing of the UAV [60], shown in Figure 19, by Innopolis University, is equipped with no less than two iris diaphragms 3. UAV 5 lands on the landing platform 1 in such a way that its legs 4 stand in the open iris diaphragms 3, then the diaphragms are closed. In this case, the edges of the diaphragm blades 3 push the legs 4 towards the geometric center of iris diaphragm 3. Electrical contacts 2 are installed at the landing site. Corresponding electrical contacts are located at the bottom of the legs 4 of UAV 5. In another version, the diaphragm blades 3 can be made of electrically conductive material and connected to the platform charger. Corresponding electrical contacts on UAV legs 4 have the form of a ring in the area of contact with the diaphragm blades. The iris diaphragms 3 are mounted with the ability to move inside the landing site, and this makes it possible to adjust the landing platform to different configurations of UAV legs.

## 5. Landing Platforms with Passive Positioning

### 5.1. Landing Platforms with Conical Funnels

The following class of landing platforms provides positioning of the UAV in the process of landing due to the passive interaction of the platform with the elements of the UAV and the transformation of the vertical movement of the UAV in the horizontal direction. The majority of these platforms are the gravitational type, i.e., the transformed motion is the UAV falling down under its own weight. Usually, the UAV falls into a funnel, on an inclined surface or on an inclined edge. Platforms of this type generally do not contain any movable elements and actuators for positioning.

#### 5.1.1. Landing Platforms with Separate Conical Funnels for Each Leg

**Figure 20:**
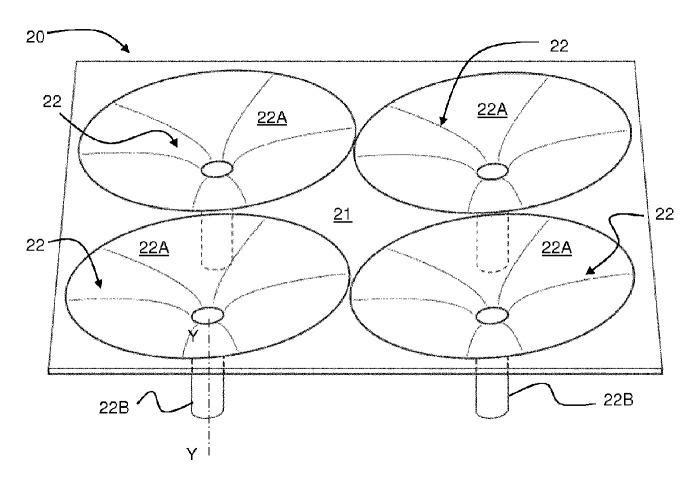
Landing platform for unmanned aerial vehicles with funnels for each leg [61] by Telecom Italia SpA. 20, landing platform; 21, flat surface; 22, centering funnels; 22A, truncated cone part of funnel; 22B, cylindrical part of funnel.

Landing platforms with a conical funnel under each UAV leg are also widespread [61,62,63,64,65]. The funnels can only carry out the positioning function [64,65] or they can also contain additional electrical contacts [61,63,66], inductors [62], or UAV fixing devices [61,66]. Figure 20 shows a landing platform for an unmanned aerial vehicle [61], which consists of a flat surface 21 and a set of centering funnels 22, embedded in this surface. The funnels 22 are arranged coaxially to the UAV legs and consist of conical 22a and cylindrical 22B parts. The UAV legs slide and move to a given position on the conical parts 22A and fall into the cylindrical parts 22B, where electrical contacts may be located for charging.

**Figure 21:**
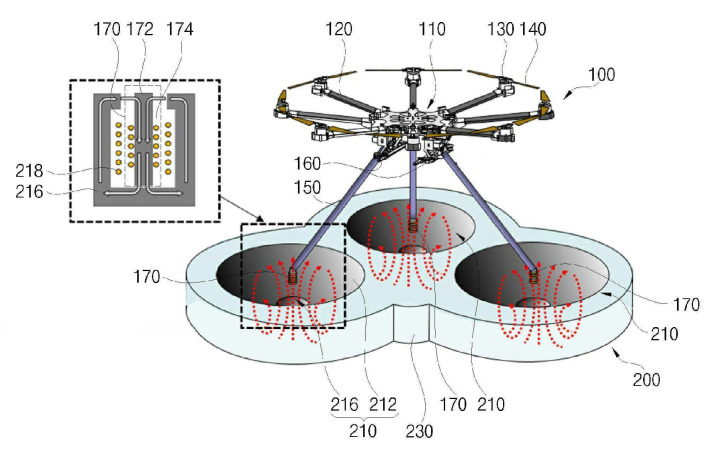
Three-phase AC-powered unmanned aerial vehicle and wireless charger [62]. 100, UAV; 150, UAV legs; 170, power receiving unit (coils); 210, landing pad; 212, conical funnels; 214, central hole (not shown); 218, feeding coils.

Figure 21 shows an unmanned aerial vehicle and a wireless charger with a three-phase power supply from the AC line [62] by the Korea Advanced Institute of Science and Technology. UAV 100 contains legs 150, ending with the power receiving unit (coil) 170. The landing platform contains three landing parts 210, consisting of conical funnels 212 and center holes 214, where the power receiving unit 170 is positioned and where the emitting coils 218 are located.

**Figure 22:**
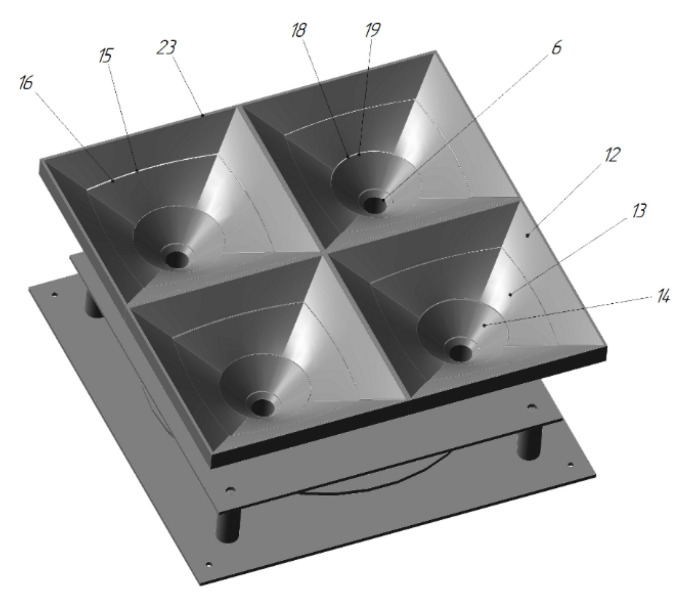
Landing platform for a UAV with funnels divided into horizontal tiers [66] by Innopolis University. 6, holes for legs; 12, 13, and 14, funnel tiers.

In the UAV landing platform [66] by Innopolis University (Figure 22), the funnels are divided into horizontal tiers 12, 13, and 14, which are folded as the UAV lands while the UAV legs pass. This results in the funnels being telescopically folded after the UAV has landed. This reduces the required height of the UAV’s legs or allows a UAV to land with a low-mounted payload on it.

The following features of the funnels as a positioning device should be considered: The angle of inclination of the funnel walls should be greater than the angle of friction of the UAV legs and funnel materials.

(1) $$\alpha >\alpha_{fr}=arctg\left(\mu \right),$$

where: α is the angle of funnel wall inclination, μ is the coefficient of friction of the UAV leg and funnel material, and $\alpha_{fr}$ is the friction angle of the leg and funnel materials. In order to reduce the angle of inclination of the funnels, the materials of the legs and funnels with a low friction coefficient are selected.

On the other hand, by changing the angle of the funnel, the lowering speed of the UAV can be adjusted. In work [61], it was suggested to make the funnel slope angle variable according to the exponential law; in work [66], it was suggested to make different slope angles in different areas of the funnel and to make larger slope angles in the upper part of the funnel, while smaller in the lower part.

Upon landing, the UAV will be successfully positioned if each leg falls into the corresponding funnel. This condition is fulfilled if the deviation of the UAV legs from the axis of the respective funnels is no greater than the following:

(2) $$X\leq R_{k},\frac{d}{2},$$

where *X* is the maximum permissible deviation of the UAV on landing; $R_{k}$ is the radius of the funnel in the upper edge; *d* is diameter of the UAV legs.

The radius of the funnel is limited by the intersection between funnels. Since the funnels for all legs have the same size, the funnels will contact each other in the middle between the legs. Obviously, the radius of the funnel should be less than half the distance between neighboring legs:

(3) $$R_{k}\leq \frac{A}{2},$$

or

(4) $$X\leq \frac{A-d}{2},$$

where *A* is the distance between the centers of the neighboring UAV legs.

For a successful landing, the UAV must achieve the landing accuracy specified in Formula 4. The funnel depth depends directly on the radius of the funnel in the upper edge and the funnel tilt angle:

(5) $$H_{k}=\left(R_{k},\frac{d}{2}\right)tg\left(\alpha \right)$$

With a large funnel radius and a large funnel inclination angle, the funnel depth can be large and require a long length of the legs.

#### 5.1.2. Landing Platforms with Overhead Cone Funnels for UAV Positioning

**Figure 23:**
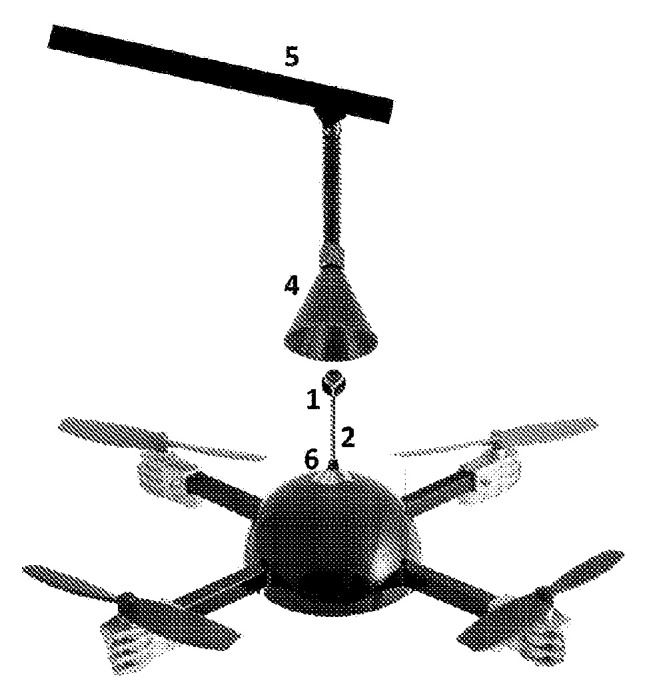
Landing platform for a vertical take-off and landing UAV with overhead cone funnel positioning [68]. 1, anchor; 2, anchor stand; 4, gripper; 5, base with open landing system; 6, housing for placing the electronic part of the control system; 7, UAV.

The works [67,68] presented landing platforms in which the UAV did not stand on legs in funnels, but was hung. For this purpose, the positioning equipment in the form of a cone was oriented upside down and had a fast griping device. The UAV had a retractable hanger in the form of a ball. The UAV flew to the landing platform from underneath and had to be suspended in the cone and climbed upwards. Conical rails pushed the ball toward the center by tilting the UAV toward the center. Tilting the UAV toward the center of the guide cone allowed the UAV to also move in the direction of the cone center, resulting in very fast positioning. The UAV took off from this landing platform, falling down like a bat after the landing platform released the grips. Figure 23 shows the landing system of an unmanned vertical take-off and landing aerial vehicle with overhead cone funnel positioning [68].

#### 5.1.3. Landing Platforms with a Conical Funnel for All Uav Legs

**Figure 24:**
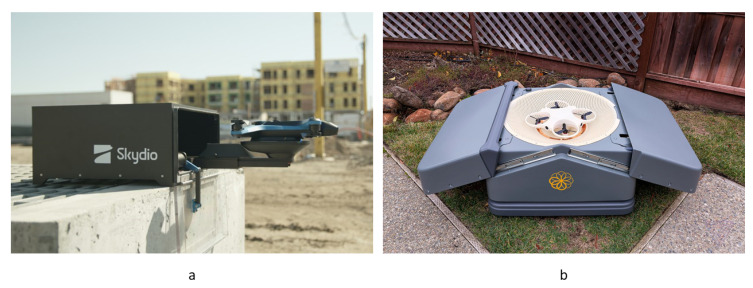
Examples of commercially available landing platforms with funnels for all legs (**a**) and whole UAV body (**b**) by (**a**) Skydio [69] and (**b**) Sunflower [70].

One funnel made for all UAV legs simplifies the design of the landing platform. This type of landing platform allows receiving UAVs with very low payload mounting. For better interaction between the funnel and the UAV legs, UAV legs could be made with a bevel or arched inwards or form a frame of an inverted truncated cone with a lower landing ring. Examples of industrial models of landing platforms for UAVs with a funnel for all UAV legs is shown in Figure 24a.

**Figure 25:**
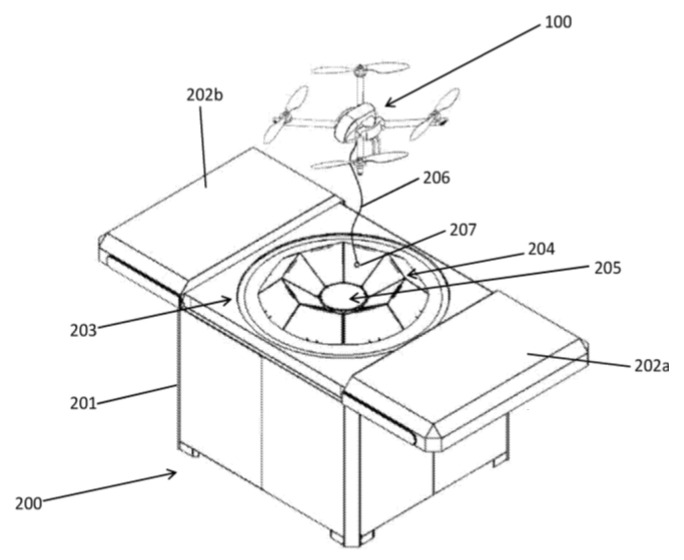
Landing platform for a vertical take-off and landing UAV with a trapezoidal segment cone for positioning [72] by Airobotics Ltd. (Petah Tikva, Israel). 100, the UAV; 203, a landing site; 204, the centering device consisting of trapezoidal segments 203; 205, centering magnetic assembly; 206, a cable; 207, a cargo.

The simplest design of such a funnel is presented in the industrial example [71] and consists of a tetrahedral funnel, the bottom of which is made according to the sizes of the UAV legs, and the side walls have an inclination for sliding the legs at landing with a deviation. Figure 25 shows a centering and landing platform for vertical take-off and landing aircraft [72] by Airobotics Ltd, which contains a landing pad 203 and a centering device 204. The centering device 204 consists of trapezoidal segments that are at the level of or below the landing pad 203 in a non-operational position and, when the UAV lands, form a multi-faceted truncated funnel. When the UAV 100 lands, it lowers the cable 206 with a payload (cargo) 207, which is gripped by the central magnet 205 of the landing platform.

The inclination of the UAV legs inside [73] or arched legs [74] in combination with a conical funnel at the landing platform allow increasing the deviation of the UAV position upon landing. In [73], contacts were made on the legs, and corresponding contacts on the funnel were made in the form of sectors. This made it possible to eliminate the necessity of the precise angular orientation of the UAV relative to the funnel. The UAV in the patent [74] by Elistair Inc. was tethered and tightened during landing by a power cable.

**Figure 26:**
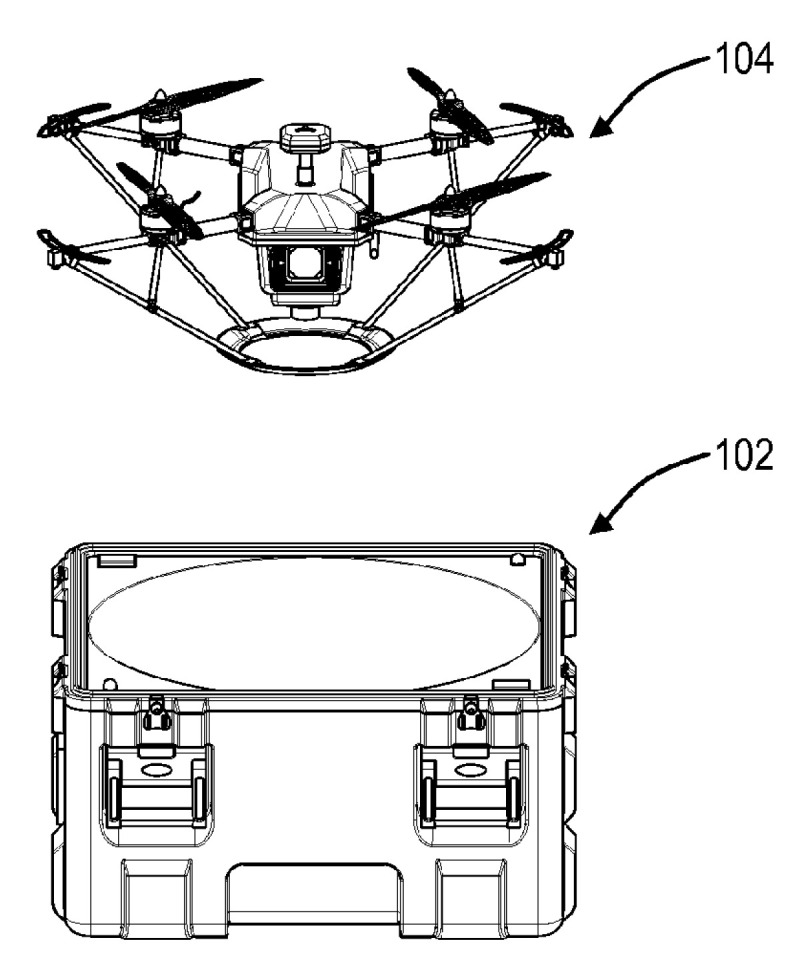
Landing station for an unmanned aerial vehicle with a funnel for all legs [76] by Skycatch Inc. 102, landing station; 104, UAV.

To facilitate the interaction between the funnels and the UAV legs, the legs can be joined by a lower ring [75]. Contacts can also be mounted on the ring to connect with the corresponding ring contacts at the bottom of the funnel. This eliminates the need for the UAV to be oriented angularly to the sides of the funnel. The UAV used with the landing station [76] by Skycatch Inc. (Figure 26) has legs inclined to the center of the UAV and joined with a lower ring. Thus, they form a truncated cone framework that interacts with the conical funnel at the landing station. The patent also covers a design in which the ring is replaced by a rectangle, and the funnel has the form of a truncated pyramid. In the patent [77], the legs are united by the lower ring and have an inclination from the center of the UAV, and a cone is installed in the center of the landing platform funnel (Figure 20). Thus, the legs’ lower ring interacts with the funnel on one side and the cone on the other when landing with the deviation of the position. This eliminates tilting moments when moving downwards. The other variant of this design is the design of the funnel and the cone as a framework. V-shaped plates, installed evenly in a circle, form a framework that interacts with the ring on UAV legs in the same way as the coaxial funnel and the pyramid in the previous example.

**Figure 27:**
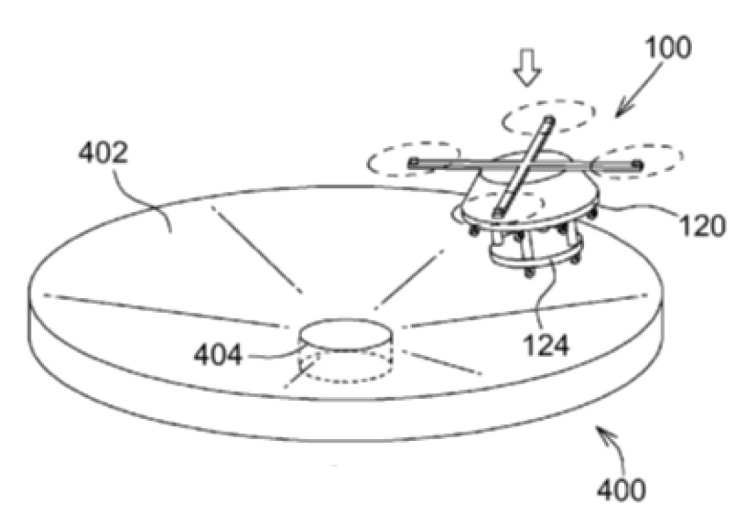
System and method of docking the UAV [78]. 100, UAV; 120, hull edge; 124, ledge; 400, landing platform; 402, landing surface (inclined surface); 404, cavity deepening.

In the patent [78] (Figure 27), the UAV legs 100 have wheels. This significantly reduces the required tilt angle of the funnel cone 402, but also makes it more difficult to hold the UAV 100 after landing. The problem was solved by introducing an additional cylindrical cavity 404 in the center of the funnel.

**Figure 28:**
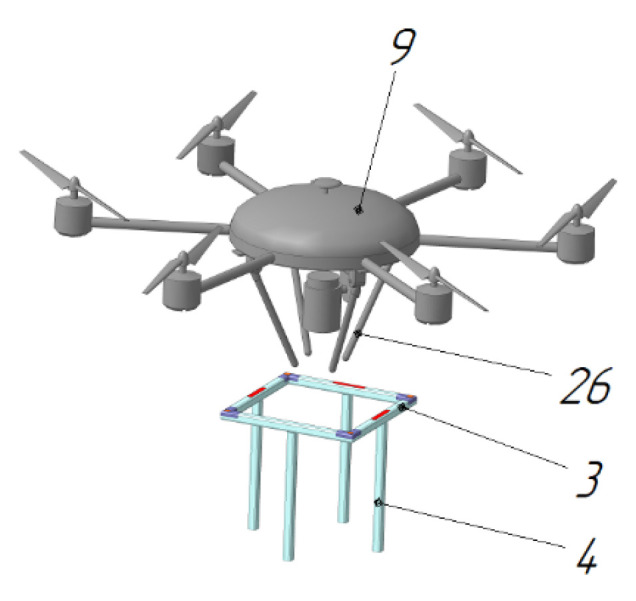
Automatic station for charging and maintenance of a multi-rotor UAV and a UAV working for it [79]. 3, landing ring; 4, landing ring supports; 9, UAV; 26, UAV chassis.

The UAV landing platform [79] (Figure 28) by Innopolis University contains a ring-shaped landing pad 3, and the chassis 26 of the UAV 9 when landing is tilted to the center, forming an upside-down cone. The interaction of the specified cone and ring upon landing causes the UAV 9 to position itself in the center of the ring. The ring can be made round, in the form of a polygon, or a star by the number of legs of the UAV. After landing, the legs 26 of the UAV are moved apart to the sides until they touch the ring and fix the UAV. Electrical contacts are installed at the landing site 3, and the corresponding contacts are located on the housing of UAV 9.

The allowable deviation of the UAV position upon landing in this scheme is determined assuming the UAV legs hit the upper part of the funnel.

(6) $$X=\frac{D-d}{2}$$

where *D* is the characteristic size of the upper plane of the funnel and *d* the characteristic size of the lower legs base.

**Figure 29:**
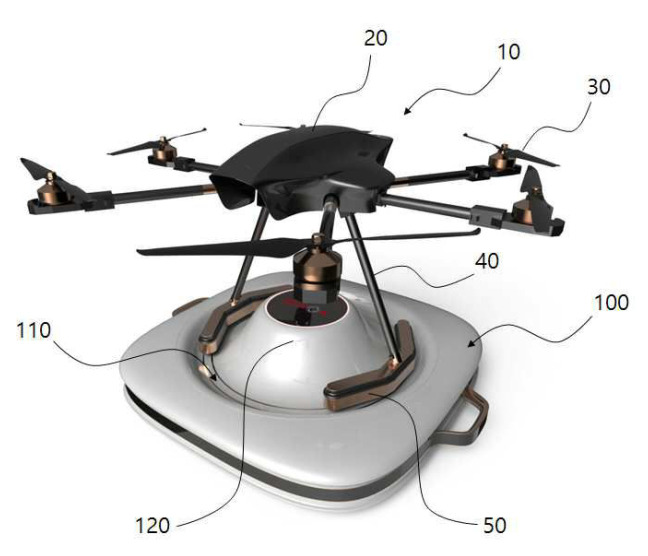
Automatic charging system with a funnel for an unmanned aircraft with ski-type legs [80]. 10, UAV; 40, legs; 50, chassis: 100, docking station; 110, landing groove; 120, central ledge.

That is why this scheme uses legs tilted to the center at landing. Work [76] presented the landing platform construction in which the funnel was replaced by a conical ledge. A similar solution was applied in the automatic charging system [80] (Figure 29). UAV 10 has two legs 40, on the ends of which the arched landing gear 50 is installed, which forms a torn circle. Docking station 100 has a central ledge 120 and a landing groove 110, which is intended for arched landing gear 50 positioning. The disadvantage of this design is the reduced clearance between the central ledge 120 of the docking station and the bottom surface of the UAV body 10, where the payload is usually mounted. However, in the case of [80], power transmission is used to charge the UAV battery by means of an inductor, which must be close to the mating coil located under the housing.

**Figure 30:**
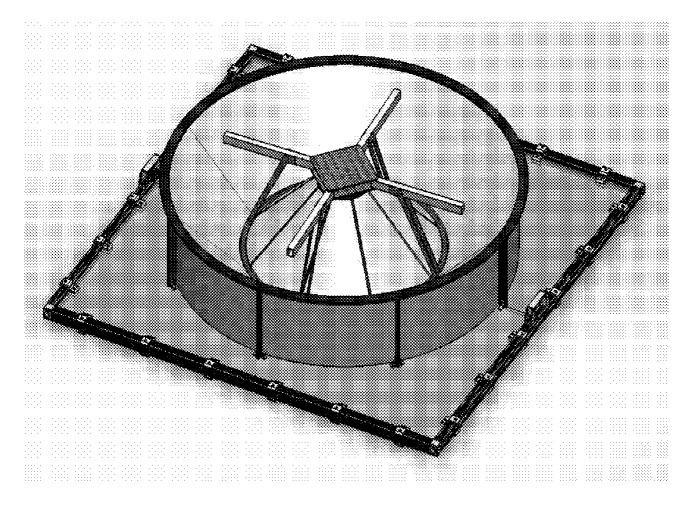
UAV docking station with a conical funnel with a protruding cone at the center for UAV legs connected by a single ring [77].

The UAV docking station in [77] (Figure 30) was a conical funnel with a protruding cone at center. The UAV legs are connected by a single ring, the diameter of which is equal to the diameter of the lowest point of the funnel. When such a UAV lands, the ring interacts with both the surface of the conical funnel and the cone. There is a variant where the funnel and the cone are replaced by frames.

**Figure 31:**
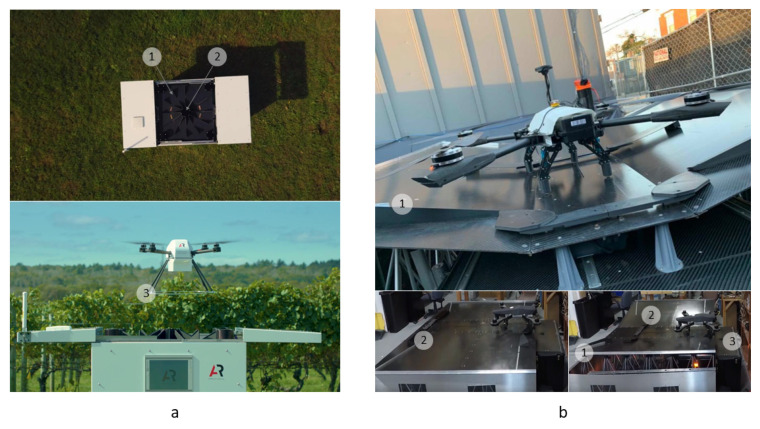
Examples of commercially available landing platforms. (**a**), Scoutbase by American Robotics, Inc. [81], landing platform with a conical funnel 1 and a protruding cone at center 2 for a UAV with a circle on the legs 3; (**b**), DroneCore by Asylon [82], a hybrid of passive and active positioning.

Figure 31a shows an industrial model created with such an approach.

#### 5.1.4. Landing Platforms for UAVs with Ski-Type Legs

**Figure 32:**
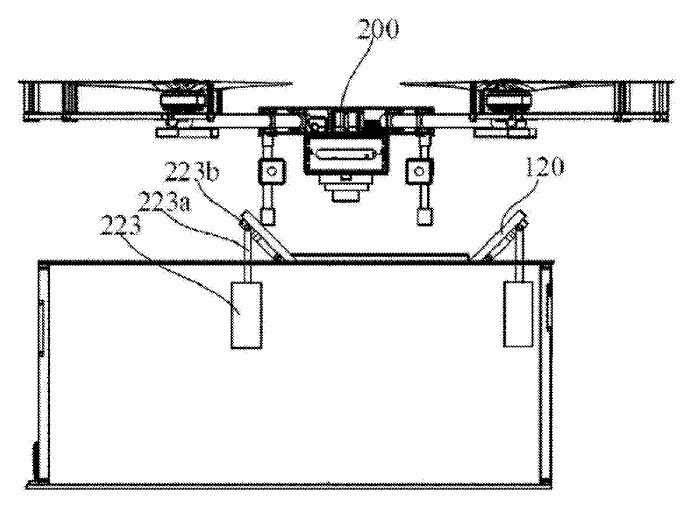
Positioning mechanism and unmanned aerial vehicle [83] by Shenzhen DJI Innovation Technology Co., Ltd. 120, rails (slope); 200, UAV.

Ski-type legs can be considered as a way of joining vertical legs or creating a stable landing gear when the UAV has only two legs. To position the UAV with ski-type legs during the landing, it is sufficient to have flat sloping surfaces at the landing platform. Positioning along the ski can be done both in the process and after the UAV has landed. The landing platform constructions, presented in patents [83,84], can be considered as landing platforms with a horizontal landing surface with lateral rails (slopes), working as centering funnels. In this case, the rails can change the tilt angle or take a horizontal position, as proposed in [83] by Shenzhen DJI Innovation Technology Co., Ltd. (Shenzhen, China) (Figure 32). In [84], the surface of the landing platform has special slots for the skis, and the electric contacts are inserted into the connectors that are installed at the ends of the skis. The platform [85] by Shenzhen DJI Innovation Technology Co., Ltd., has a funnel of a rectangular shape, the side walls of which interact with the UAV skis, providing positioning. The landing platform in [86] has a protruding rectangular pyramid positioning device, on top of which there is a hatch for receiving the payload from the UAV.

#### 5.1.5. Landing Platforms with Funnels for the Whole UAV Body

**Figure 33:**
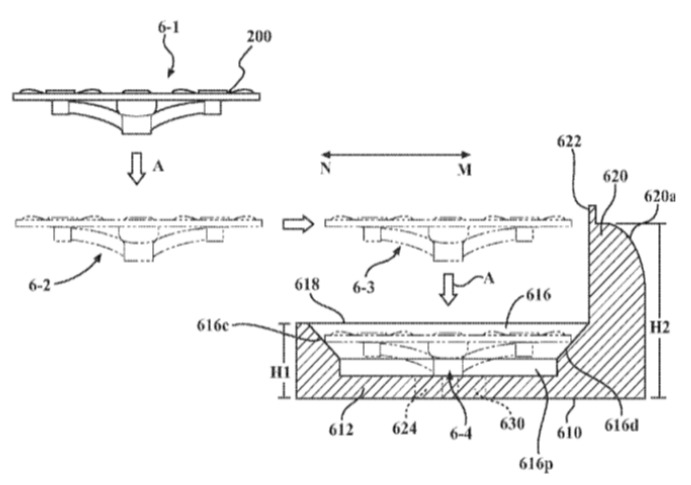
System for the transport, deployment, and docking of UAVs mounted on ground vehicles with a funnel for the whole UAV body [87] by Toyota Motor Corp. 200, UAV; 610, landing platform body; 616, centering funnel; 616r, funnel; repeating the body of the UAV; 618, inlet opening.

Another approach for positioning the landing UAV involves the interaction of the UAV body with the landing platform. In such devices, the UAV and the landing platform are developed and used together. The funnel in the lower part follows the shape of the UAV body, serves as a base for it, and expands upwards [87,88]. Often in such constructions, the landing platform is a case for UAV storage between flights and during transportation, as proposed in [87] by Toyota Motor Corp (Figure 33).

**Figure 34:**
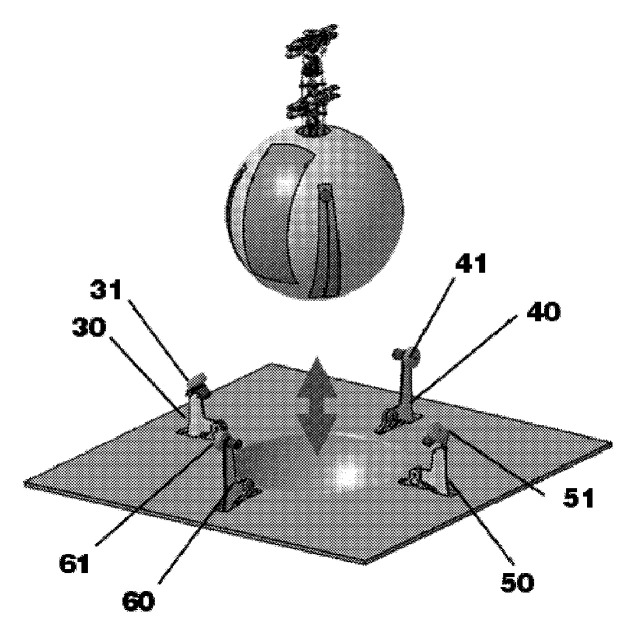
The unmanned aerial vehicle with spherical housing and the landing platform for it [89]. 30, 40, 50, and 60, levers; 31, 41, 51, and 61, drive wheels.

The central hull of the unmanned aerial vehicle in [89] (Figure 34) is made in the form of a sphere, and in turn, the landing site is made in the form of a reciprocal spherical depression. The lever grips the four sides of the spherical depression to hold the UAV. At the ends of the lever,s there are drive rollers that can be rotated to align the UAV. This design can receive and launch the UAV regardless of the inclination of the landing platform.

**Figure 35:**
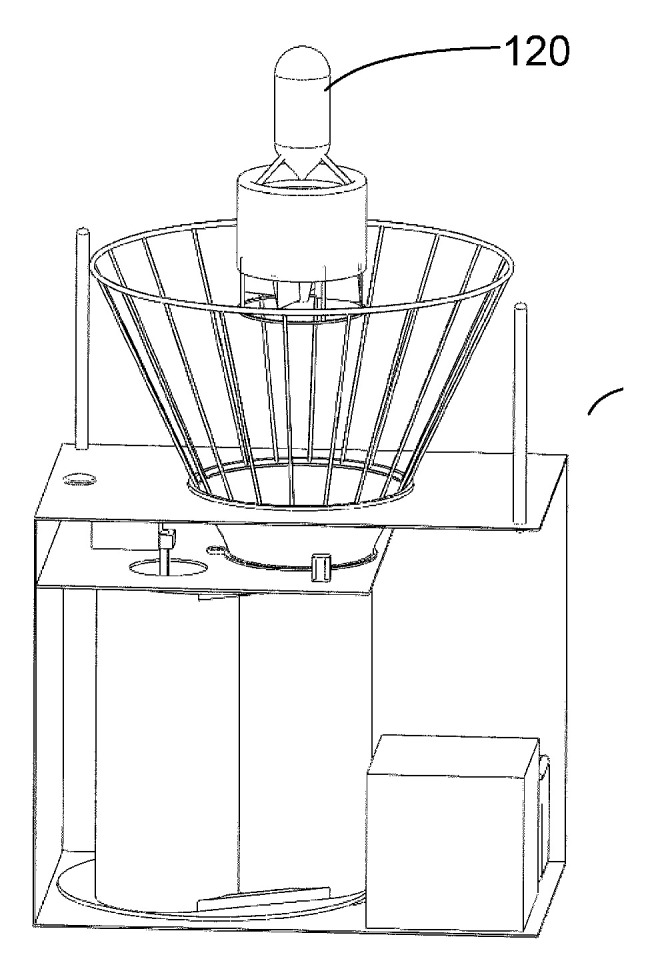
Ducted fan unmanned aerial vehicle docking station [90].

Ducted fan unmanned aerial vehicle docking station [90] Figure 35 uses a funnel made of bars in the form of a frame. The UAV itself has a central propeller, which is installed in the diffuser (ducted fan). The skeleton funnel eliminates the effect of air flow from the UAV on the landing and positioning process.

An example of the industrial model of a landing platform for UAVs with a funnel for whole UAV body by Sunflower Labs Inc. (Birmensdorf, Switzerland, San Francisco, CA, United States) is shown in Figure 24b.

### 5.2. Landing Surfaces in the Form of Closed Contours

The next type of landing platforms providing the positioning of a UAV uses the landing supports (legs) of a UAV of a horizontal type installed as beams and a landing surface in the form of a various-height closed contour.

**Figure 36:**
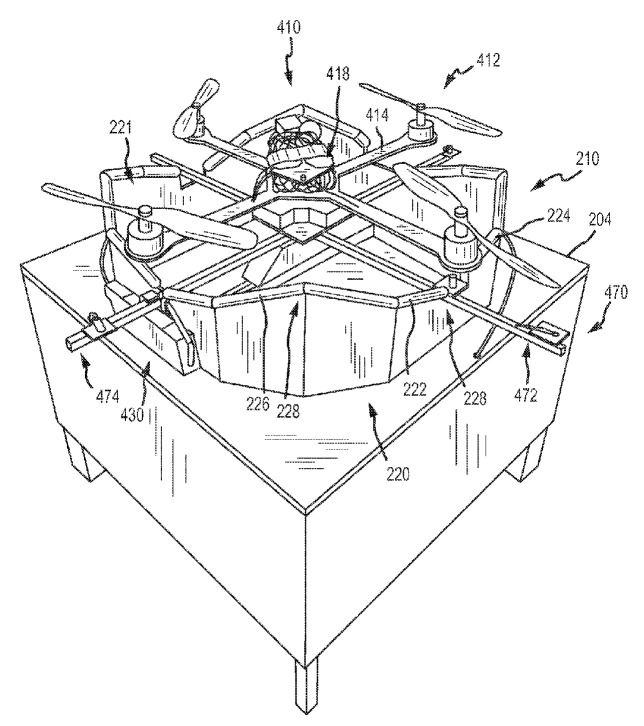
System for the autonomous docking and charging of an unmanned aerial vehicle with a closed contour for positioning [91] by ETHZ and Disney Enterprises Inc. 222, 224, and 226, receiving surfaces (inclined); 228, tops of receiving surfaces; 410, UAV; 414, rotor supports (beams); 474, levers (horizontal supports).

In the system of autonomous docking and charging of an unmanned aerial vehicle in Figure 36 [91] by ETHZ and Disney Enterprises Inc., the landing site is made in the form of a corona, and the landing supports of the UAV are located crosswise at the bottom. There are grips to hold the UAV and electrical contacts in the cavities of the corona contour. The UAV lands on receiving surfaces 222, 224, and 226 with horizontal supports 474 and slides on these surfaces to the lower point, centering relative to the center axis of the corona, where it is gripped and an electrode for charging is connected.

**Figure 37:**
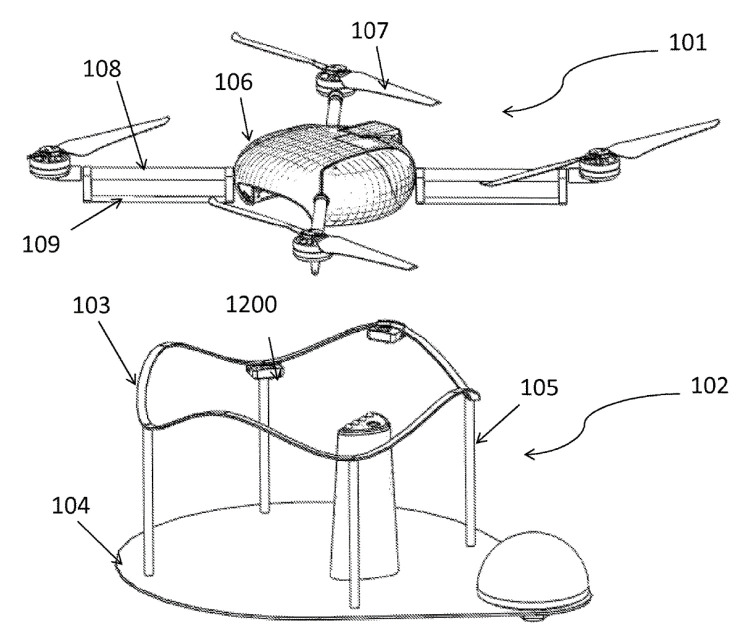
UAV landing system with a closed contour for positioning [92] by Kespry Inc. 101, UAV; 102, landing platform; 103, track; 104, base; 105, stands; 107, propeller of the UAV; 108, beams of the UAV frame; 109, horizontal landing supports.

In the system for the landing of an unmanned aerial vehicle in Figure 37 [92] proposed by Kespry Inc., the closed contour of the landing site (track 103) is made of ribbon elastic material and has periodic lifting and lowering areas with lower points mounted on supports 105. UAV 101 lands on track 103 with horizontal supports 109 and slides down the track, which results in the UAV aligning with the center of the track.

**Figure 38:**
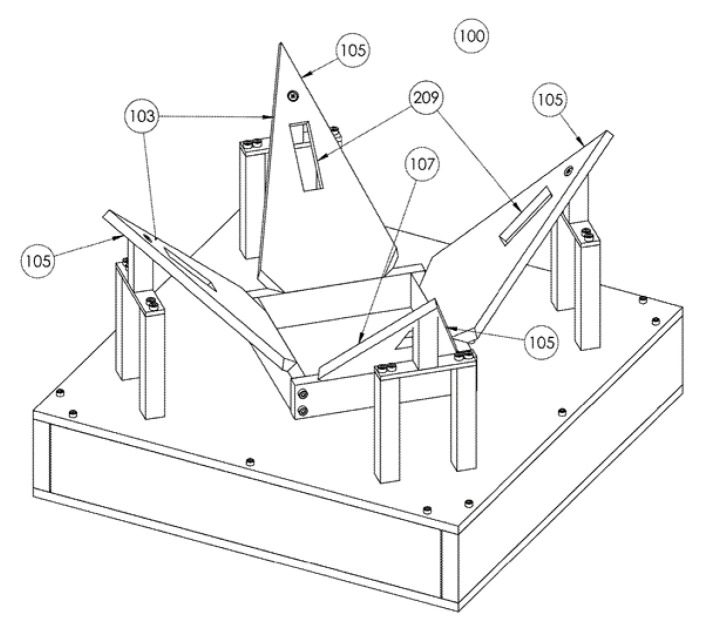
Aircraft docking station with a closed contour for positioning [93] by American Robotics Inc. 100, docking station; 103, centering system; 105, ledges.

The aircraft docking station by American Robotics Inc. in Figure 38 [93] has circularly arranged triangle-shaped inclined ledges. When the UAV lands, the edges of the triangles interact with the rays of the UAV and position it. The protrusions have special rotary latches to fix the UAV after landing.

**Figure 39:**
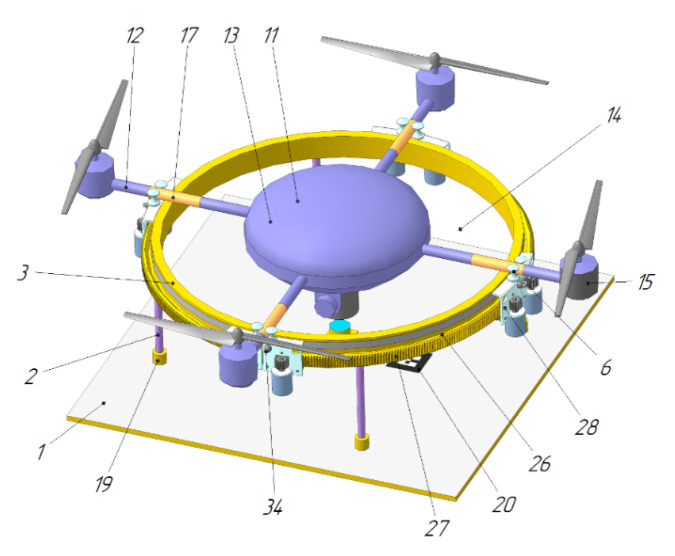
Landing platform for a vertical take-off and landing UAV with an active ring for positioning [95] by Innopolis University. 3, landing site ring; 6, pushers; 11, UAV; 12, UAV beams; 28, pusher actuators.

In work [94], the ring-type landing surface also has height variations and contains a gripping device to hold the UAV after positioning. In the platforms of [91,92,94], the landing sites are made with variable heights and are located in the area of the lifting propellers. In order to prevent the propellers from touching, the UAVs in [91,92] have landing legs located below the UAV beam. In the patent [94], the UAV lands on beams, but it is specified that the friction of the beam of the UAV and the circular contour of the landing site should have a very low coefficient. The landing platform for the vertical take-off and landing UAV Figure 39 [95] by Innopolis University has a landing site 3, made also in the form of a closed contour, and UAV 11 lands with the beams 12. However, this design has an active device for positioning and fixing the UAV after landing (gripper), and the landing pad 3 is horizontal. The pushers 6 of the gripper move independently of each other on a closed surface, catching the beams 12 of the landed UAV 11, and then perform its positioning. This landing platform is equipped with an active UAV position and orientation tracking system, which allows moving the gripper in the area where UAV beams 12 are expected to be located and to fix the UAV quickly after landing. The speed of this design does not depend on the accuracy of the UAV landing and, therefore, should be superior to other platforms when the UAV is landed in difficult landing conditions [91,92,94].

To assess the required accuracy of the UAV landing on this type of platform, it should be considered that the success of the landing is primarily determined by hitting the UAV body inside the ring. The body of the UAV could move radially inside the hole of the landing site by the value of:

(7) $$X_{1}=\frac{D+d}{2},$$

where *D* is the diameter of the hole in the landing site and *d* the diameter of the UAV body. If the hull of the UAV is not round in shape, diameter *d* is determined by the protruding elements of the hull, which will affect the inner surface of the landing site and limit the displacement from the center of the ring. This is the permissible deviation of the UAV landing.

On the other hand, the UAV’s rays can move around the landing site within their lengths by a magnitude of:

(8) $$X_{2}=\frac{D_{1}+d_{1}}{2},$$

where d1 is the maximum diameter of the UAV beams and D1 is the outer diameter of the landing pad. This diameter determines the restriction on movement of the motor mounted at the end of the UAV beam; D2 is the mean diameter of the landing surface ring between the diameters *D* and D1. The optimal dimensions of the ring-shaped landing site are such that when the hull of the UAV is located at the inner edge of the landing site, the UAV engine closest to the center of the landing site is located at the outer edge of the landing site ring. This condition is fulfilled if the middle diameter of the landing ring is the same as the length of the beams.

Main types of positioning devices in UAV landing platforms, covered above, are presented at a glance in the Table 1.

### 5.3. Combined Positioning Devices

The combination of different positioning devices on a landing platform makes it possible to simplify the design or to increase the positioning accuracy and speed by taking advantage of each device. In addition, these devices can operate both in parallel and in sequence.

In the landing platform [60] in Figure 19, the main positioning device is an iris diaphragm. For the expansion of an admissible deviation upon landing, there are funnels over iris diaphragms. This landing platform thus has passive and active positioning devices installed in sequence.

**Figure 40:**
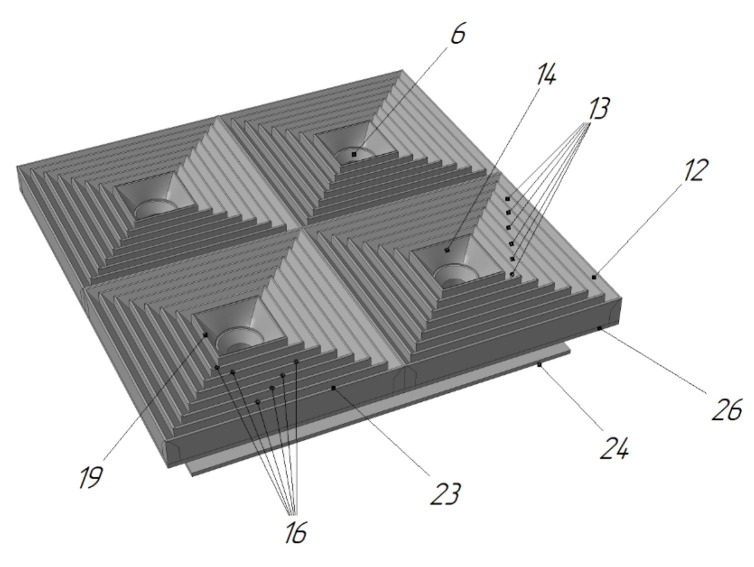
Landing platform for a UAV with a running wave funnel as an example of combined active and passive positioning [66] by Innopolis University. 13, funnel tiers’ 16, funnel tier tops.

The landing platform for the UAV in [66] represents a variant of the design that implements a convergent running wave (see Figure 40). The device contains many tiers of the funnel 13, which are set in such a way that the upper parts 16 form the landing plane. The UAV legs have such size that they are always on two adjacent tiers. After the UAV has landed, the tiers rise up and fall back in sequence starting from the outer tiers. When the tier is raised, the support above it will be pushed to the center. In this way, the wave that moves towards the center of the UAV positions it. The active vertical movement of the funnel tiers results in passive positioning. This design can be realized either for an individual leg or for a group of legs.

The centering and landing platform [72] by Airobotics Ltd. also has the possibility to change the funnel angle or transform it into a plane and thus influence the process of positioning (Figure 15). The DroneCore landing platform by Asylon [82] shown in Figure 31b is an industrial example of a hybrid positioning device with a 2D funnel 1 with a controllable angle and an additional pusher 2 to move the UAV toward the battery replacement device 3. In designs of landing platforms presented in patents [83,84], lateral slopes have an actuator allowing one to change the angle of their slope and thus to influence the positioning process.

In the unmanned aerial vehicle with a spherical housing and a landing platform for it [89] in Figure 34, the UAV is additionally pressed after landing with levers 30, 40, 50, and 60 and moved to the required direction with actuated wheels 31, 41, 51, and 61 (Figure 23). This solution allows holding the UAV vertically, even after landing on an inclined landing site.

## 6. Non-Standard Landing Platforms

**Figure 41:**
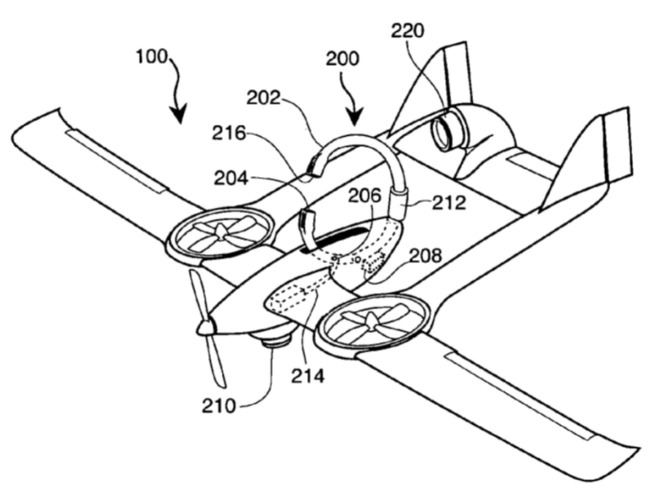
Charging from the power supply line [96]. 100 UAV; 202, magnetic core; 212, winding; 204, movable part of the magnetic core; 216, surface of the interfacing of parts of the core,

The landing platform for the UAV can be a variety of devices, sometimes extraordinary at first sight. For example, in the patent [96] (Figure 41), a power line wire is chosen as a landing device.

UAV 100 is hooked on a wire of an electric line by means of the magnetic core 202 with a winding 212. The core 202 for this purpose is made open with a movable part 204. After the capture of a wire of a power line, the hook is closed in a ring. The resulting closed core has a wire of the power line as a primary transformer coil and a secondary winding 212 that supplies the battery charger. The similar problem is solved by the device [97], which grips the power line wire from above.

**Figure 42:**
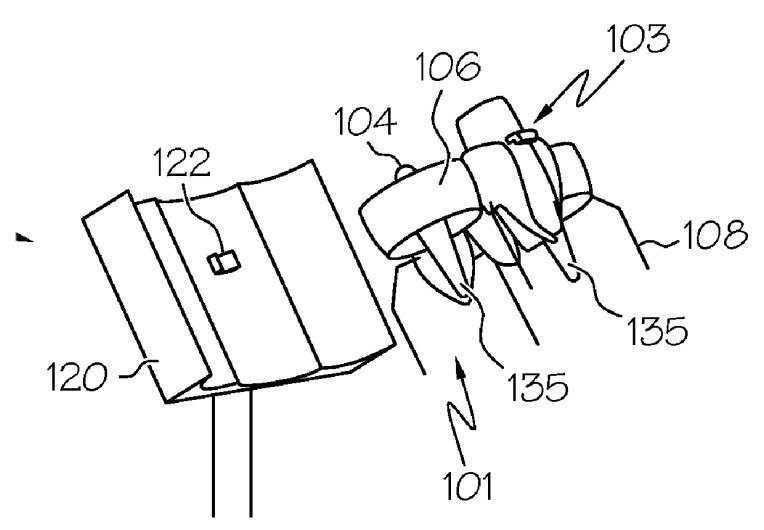
UAV launch and landing system [98] by Honeywell International Inc. 101, UAV; 120, capture plate; 122, carrying bracket.

In a UAV launch and landing system [98] by Honeywell International Inc., shown in Figure 42, the UAV is landed on the support bracket 122, which is mounted on a special profile tray 120 that provides the gas-dynamic pull of the UAV at the moment of landing. The UAV is driven by two impellers 106 installed in parallel.

**Figure 43:**
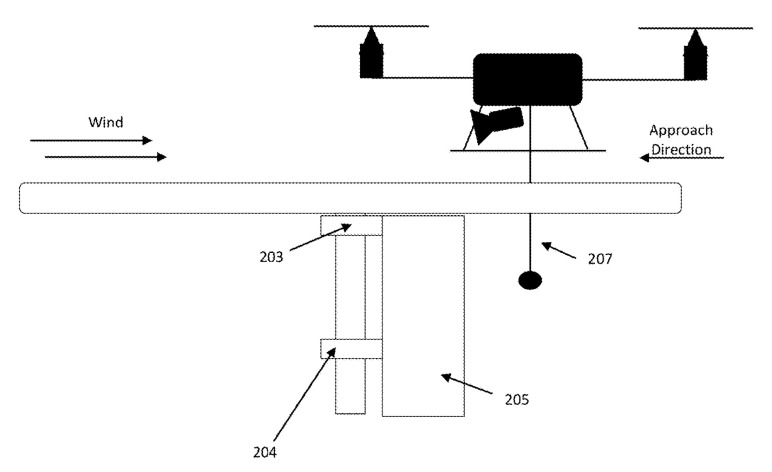
Unmanned aerial vehicle reload/refueling station [99]. 203, free rotation clutch 205; cable grab mechanism; 207, cable.

The reloading/refueling station for unmanned aerial vehicles in [99] (Figure 43) has a rotatable landing area with a sector cut, which is always oriented toward the windward side. The cargo is hung on the UAV by a special cable 207. The UAV arrives for landing on the windward side, and when flying over the landing site, the cable enters the sector cut. The landing platform detects the presence of the cable and pulls the UAV into the landing area.

## 7. Conclusions

Positioning and fixing of a UAV at the landing site are necessary operations for recharging or replacing batteries, loading and unloading the UAV, or performing other operations in automatic mode.

Both active devices and mechanisms, as well as passive elements are used for positioning. Active devices provide positioning by pushing the chassis or other elements of the UAV after the UAV has landed on the landing site. They are used both for simple UAV displacement in the direction of the pusher’s movement and for moving the UAV simultaneously in two directions or for more complex movements. Passive methods of positioning of the UAV, providing the transformation of the vertical movement of the UAV in the horizontal direction by the interaction of the elements of the UAV with the elements of a landing site have found wide application. These techniques include funnels, inclined surfaces, or edges. These elements can interact with each of the UAV legs separately or with a group of UAV legs, the UAV hull, UAV beams, etc.

The landing platforms are distinguished by a great variety of designs and the number of publications on this topic over the past three years. This shows that at the moment, there are strong development and establishment of automatic landing platforms with UAV positioning devices on the landing site. Future tendencies in the field of UAV landing stations include reducing the size of the landing station with increased accuracy of the UAV landing and the UAV dimensions themselves. Other trends relate to developing special designs of positioning and fixing devices for challenging landing conditions, on moving ground or marine vehicles, or with wind. Such landing stations could have the ability to catch the UAV with a non-horizontal orientation, active UAV tracking, and adjustment of the landing platform systems to the current position, orientation, and speed of the UAV. The main trend in the application of landing stations is the expansion of their functionality regarding cargo unloading and usage as vending machines. Furthermore, information systems to support the application of landing stations for other tasks such as monitoring and mapping are being developed, including the group application of multiple UAVs with landing stations and heterogeneous groups with UAVs and ground vehicles. Landing stations serving multiple UAVs are being developed, as well as UAV cassette launching systems. Another possible direction of landing stations’ development will be the adaptation for servicing large VTOL planes, which are actively evolving now.

## Figures and Tables

**Table 1:** Main positioning device types in UAV landing platforms at a glance.

**1 No positioning**											
**Pros**: simple and robust, because there are no actuators and mechanical moving parts **Cons**: the UAV and its electrical contacts are not fixed at the landing site; the UAV may move out of position and lose its electrical contact, or it may land on one electrical contact with opposing polarity contacts											
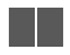				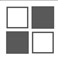				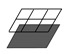			
1.1 Landing site divided into two parts with rectangular contacts **Pros**: simple				1.2 Alternating electrodes **Pros**: the large number of contacts eliminates the requirement for the accuracy of UAV landing				1.3 Vertical separation **Pros**: the UAV is charged regardless of the accuracy of landing; different contacts are separated			
**2 Active positioning**											
**Pros**: fixes the UAV (in the wind, on a moving platform); operation is not affected by the accuracy of the UAV’s landing (in the area of the landing site) **Cons**: has moving parts and actuators; positioning and fixing should be automatically controlled											
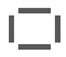			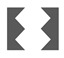			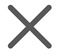			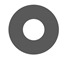		
2.1 Parallel pushers **Pros**: are suitable for use with UAVs of different sizes			2.2 Profile (V,W) pushers **Pros**: reducing the number of pushers; positioning both in the direction of the movement of the pushers and orthogonal **Cons**: is suitable for use only with UAVs of a given size and number of legs			2.3 Rotating pushers **Pros**: reducing the number of pushers **Cons**: is suitable for use only with UAVs of a given size and number of legs; does not fix the UAV			2.4 Diaphragms **Pros**: could be configured for UAVs of different sizes and number of legs; quick fixing of the UAV		
**3 Passive funnels**											
**Pros**: simple; no actuators or moving devices; passive positioning simultaneously with landing **Cons**: usually has a small landing site; requires better landing precision; could be unreliable in difficult landing conditions (on a movable platform, under vibration, wind, landing with horizontal speed or with an angle)											
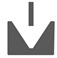			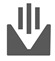			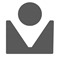			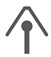		
3.1 Under each leg **Cons**: suitable only for a given UAV leg configuration; needs a high payload mount			3.2 One for all legs Most used type **Pros**: suitable for a UAV with a low payload mount			3.3 For the whole UAV body **Pros**: no legs needed; the landing platform could be used as a storage container			3.4 Overhead **Pros**: self-stabilization (centering) during docking; possibility of non-standard installation (on street lights, etc.)		
**4 Closed contours**											
**Pros**: Simple; no actuators or moving devices for positioning; passive positioning simultaneously with landing; simplified UAV construction (no UAV legs are needed); the UAV center of mass is below the fixation point; could be used with a UAV with low payload mounting; lower landing precision is usually required (comparing with funnels) **Cons**: UAV modification is usually needed to land on a closed contour											
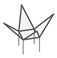				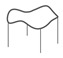				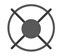			
4.1 Corona				4.2 Track				4.3 Circle **Pros**: Reversed landing sequence: fix first and positioning after			

## Abbreviations

The following abbreviations are used in this manuscript:

MDPI	Multidisciplinary Digital Publishing Institute
DOAJ	Directory of open access journals
UAV	Unmanned aerial vehicle
VTOL	Vertical Take-Off and Landing
AC	Alternating current
